# Evolution of Chemical, Structural, and Mechanical Properties of Titanium Nitride Films with Different Thicknesses Fabricated Using Pulsed DC Magnetron Sputtering

**DOI:** 10.3390/ma17246067

**Published:** 2024-12-12

**Authors:** Wei Mao, Runze Qi, Jiali Wu, Zhe Zhang, Zhanshan Wang

**Affiliations:** 1 MOE Key Laboratory of Advanced Micro-Structured Materials, Institute of Precision Optical Engineering (IPOE), School of Physics Science and Engineering, Tongji University, Shanghai 200092, China; 2111188@tongji.edu.cn (W.M.); 2010552@tongji.edu.cn (J.W.); zzgight@tongji.edu.cn (Z.Z.); wangzs@tongji.edu.cn (Z.W.); 2 Zhejiang Tongyue Optical Technology Co., Ltd., Taizhou 318013, China

**Keywords:** titanium nitride, roughness, microstructure, chemical composition, intrinsic stress

## Abstract

Considering the application of titanium nitride (TiN) films as a release layer in producing Wolter-I X-ray telescope mirror shells by the electroformed nickel replication (ENR) technique, this research pays attention to the influence of nanometer-scale thickness variation in the microstructure and physical properties of TiN films deposited by the pulsed direct current (DC) magnetron sputtering method. This topic has received limited attention in the existing literature. TiN films (9.8 nm to 42.9 nm) were fabricated to comprehensively analyze the evolution in microstructure, depth distribution of elements, surface morphology, and intrinsic stress. With increasing thickness, TiN transitioned from amorphous to (200) and (111)–(200) mixed-oriented crystallization, explaining inflection points in the increasing roughness curve. Elements (C, N, O, Si, and Ti) and chemical bond proportions (Ti-N, Ti-N-O, and Ti-O) varied with film depth, and the fitting of film density can be optimized according to these variations. Crystallite size increased with thickness, which led to a reduction in intrinsic stress. It is evident that as film thickness increases, TiN forms a stable crystal structure, thereby reducing intrinsic stress, but resulting in increased roughness. Considering the impact of changes in thin film thickness on physical properties such as roughness, crystallinity, and intrinsic stress, a TiN film with a thickness of approximately 25 nm is deemed suitable for application as a release layer.

## 1. Introduction

The electroformed nickel replication (ENR) technique has been widely used in the preparation of Wolter-I X-ray telescope mirror shells for many X-ray astronomy missions, such as Swift-XRT and XMM-Newton [1,2]. The release layer deposited on the mandrel plays an important role in the ENR process by helping to reduce stress during separation, thereby producing replicas that retain the shape of the mandrel. Traditional metal monolayer films (such as Au and Ir) adhere to the surface of the telescope mirror shell during separation, acting as both the release layer and the reflecting layer [3]. This technology can also be used in nested neutron-focusing optics [4]. Nevertheless, using these two materials tends to increase the roughness of the mandrel slightly during the replication process, so the mandrel should be re-polished after multiple replications. Additionally, since these release layers are removed along with the telescope mirror shells during separation, they must be re-deposited after each replication. Since the mixed covalent Ti–N and metallic Ti–Ti bonding exists and charge transfer from Ti to N atoms can be observed in titanium nitride (TiN) [5], this proves that TiN contains few free electrons and is not easily combined with other substances. The advantage of using TiN films as the release layer is that it remains on the mandrel to achieve multiple replications while maintaining surface quality, and the reflecting layer deposited on TiN remains on the surface of the telescope mirror shells, so different monolayer or multilayer films can be selected as the reflecting layer [6]. Furthermore, X-ray multilayer films offer higher selectivity, absorption capacity, wavelength range coverage, and control over relative phase compared to monolayers. Therefore, TiN is an ideal material for the release layer.

In addition, TiN thin films have extensive applications, such as in metal material processing and microelectronics industries, due to their excellent thermal stability, electrical conductivity, hardness, and adhesion [7]. The microstructures of TiN thin films are highly related to their functional properties. Therefore, existing studies on TiN pay more attention to the impact of deposition parameters on microstructural behaviors [8]. Lu et al. found that substrate bias can affect the resistivity and hardness of TiN films [9]. For TiN films, an optimum resistivity (minimum) of 19.5 μΩ-cm and a hardness (maximum) of 31.5 GPa were achieved at the 100 V substrate bias. Chawla et al. found that TiN films with micron-scale thickness deposited under an Ar + N_2_ atmosphere initially exhibited a (200) preferred orientation, which subsequently changed to a mixed (111)–(200) orientation with increasing deposition time at 500 °C [10]. Ali et al. found that surface roughness mainly depends on the properties of the substrate and its surface, deposition time, coating thickness, substrate temperature, and bias voltage [11]. This research shows that by decreasing the deposition time from 120 to 30 min, the values of surface roughness parameter Rq gradually reduced from 0.94 to 0.38 μm. The N/Ti atomic ratio of TiN films prepared by DC magnetron sputtering was about 0.5 in the study by Abdallah et al., and the roughness and crystallization changed with a thickness of hundreds of nanometers [12]. In this study, the roughness increased from 1.7 nm to 2.5 nm with an increase in the deposition time from 15 min to 20 min (with an increase in the thickness to 500 nm for 20 min).

TiN thin films are deposited by pulsed DC magnetron sputtering to obtain high-quality thin films. Yeh et al. demonstrated that TiN coatings deposited using pulsed DC magnetron sputtering exhibit enhanced hardness and superior electrical resistivity compared to those produced by conventional DC sputtering [13]. The pulsed DC technique increases both the ion flux ratio and the kinetic energy of bombarding ions, promoting the formation of denser films with finer grain structures and elevated levels of compressive residual stress than the traditional DC method [14].

Since the roughness of the telescope mirror shells is consistent with the roughness of the release layer, we need to keep the roughness of TiN films down to the sub-nanometer scale. This implies a need to investigate TiN films of tens-of-nanometer-scale thicknesses. Also, to avoid degradation of the release layer during separation, high-quality TiN thin films fabricated by pulsed DC magnetron sputtering are desirable [6]. However, according to Table 1, far too little attention has been paid to the influence of tens-of-nanometer-scale thickness variation in the microstructure and physical properties of TiN films fabricated by pulsed DC magnetron sputtering. With the exception of Ref. [15], where a TiN film with a thickness of 7 nm was deposited using the traditional DC sputtering method, all other TiN films listed in Table 1 are relatively thick (greater than 150 nm). Prior to our study, limited research focused on the deposition of relatively thin TiN films with pulsed DC sputtering. In contrast, our work utilizes the pulsed DC sputtering technique to deposit very thin TiN films (10–40 nm), which are significantly thinner than those reported in other studies utilizing pulsed DC sputtering, as seen in Table 1. Therefore, our study is essential for exploring the microstructure, surface morphology, and intrinsic stress variation as a function of thickness in pulsed DC TiN films within the tens-of-nanometer scale.

In this paper, the evolution of TiN films with thicknesses varying from 10 to 40 nm fabricated by pulsed DC magnetron sputtering were explored. Based on the application of TiN in the space field, our research investigated the roughness and intrinsic stress of TiN thin films with varying thicknesses. The growth characteristics of TiN thin films were analyzed through the changes in the microstructure and composition of the film with depth by XRD, XPS, and AFM.

## 2. Experimental

TiN films with varying thicknesses (as shown in Table 2) were deposited by using pulsed DC magnetron sputtering. In the experiment, 50%Ar + 50%N_2_ was used as the working gas, and the pressure of the working gas was 0.399 Pa during the deposition procedure. The base pressure was reduced to 6.0 × 10^−5^ Pa before the sputtering process. A Ti target with dimensions of 508.0 mm long, 38.1 mm wide, and 3.2 mm thick was used, with a target-to-substrate distance of 100 mm, and the substrate temperature was room temperature. For pulsed DC magnetron sputtering, the power supply operated at a constant power of 400 W, with a pulse frequency of 40 kHz, a duty cycle of 10%, and a voltage of approximately 370 V. The TiN thin films were deposited on super-polished silicon wafers (20 mm × 20 mm, thickness 0.5 mm) and round quartz substrates (diameter 30 mm, thickness 1 mm). Silicon wafers were used to measure film thickness, roughness, and crystal orientation. Round quartz substrates were selected to measure intrinsic stress. By performing a 1 μm × 1 μm atomic force microscope scan, the root mean square (RMS) roughness of the silicon wafer was found to be less than 0.2 nm.

A Bruker D8 Discover X-ray diffractometer (Bruker, Madison, WI, USA) with a Cu-Kα source (λ = 0.154 nm) was used to determine the layer thickness and crystal orientation of these samples through grazing incidence X-ray reflectometry (GIXRR) and grazing incidence X-ray diffraction (GIXRD).

X-ray photoelectron spectroscopy (XPS) depth profile analysis was performed with a Thermo Scientific K-Alpha+ spectrometer (Thermo Fisher Scientific, Waltham, MA, USA) to demonstrate the variation in the chemical composition of a TiN film of 42.9 nm thickness with depth. The sample was etched with 1 keV Ar^+^ ion from the surface to the silicon substrate with an etching area of 2 mm × 2 mm. Then, an X-ray spot of 400 μm was used to acquire the high-resolution spectrum. In XPS depth profile analysis, the binding energy curves of C 1s, N 1s, O 1s, Si 2p, and Ti 2p were recorded. These spectra were then calibrated against peak C 1s (284.8 eV).

The morphology was measured by using atomic force microscopy (AFM) on a Bruker Dimension Icon system (Bruker, Madison, WI, USA). We conducted tests on image areas of 10 μm × 10 μm, 5 μm × 5 μm, and 1 μm × 1 μm, respectively, with each AFM image consisting of 256 × 256 lines of pixels. The surface fluctuations in AFM images were transformed into power spectral density (PSD) functions using Fourier analysis.

The intrinsic stress of the TiN films was calculated using Stoney’s formula based on the curvature radius difference [20]. The curvature radii of the quartz substrates before and after fabrication were measured by a ZYGO interferometer (λ = 632.8 nm) (Zygo, Middlefield, CT, USA).

## 3. Results and Discussion

### 3.1. GIXRR Fitting—Film Thickness and Roughness

**Figure 1:**
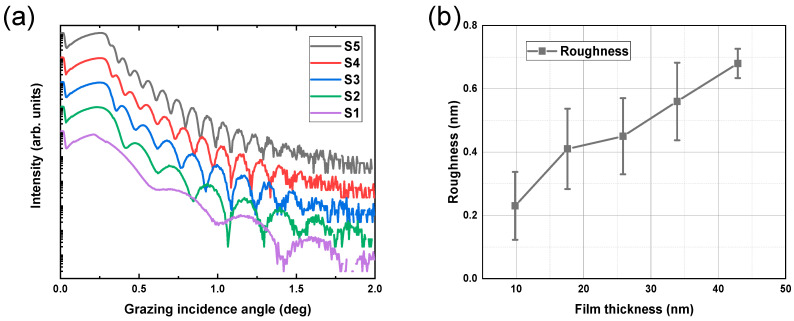
(**a**) GIXRR measurements of TiN thin films at a wavelength of 0.154 nm. TiN thin films were prepared at varying deposition times. (**b**) Surface roughness of TiN thin films with varying thicknesses fitted by using a genetic algorithm.

Figure 1a shows the GIXRR curves of TiN thin films prepared at proportionally increased deposition times, which range from 7.5 min to 37 min. The Kiessig fringes of all five GIXRR curves are different, indicating different film thicknesses for each corresponding sample. The film thickness and roughness could be obtained by using genetic algorithms [21] to fit the curves in Figure 1a. According to the fitting results presented in Table 2, the TiN film thickness of five samples ranged from 9.8 nm to 42.9 nm. As expected, an increase in deposition time led to a proportional increment in film thickness, demonstrating consistency with design expectations.

Table 2 also provides information on the surface roughness of these thin films, and the surface roughness of all samples is greater than that of the silicon substrate itself. Furthermore, it can be observed from Figure 1b that as fitted film thickness increases, so does the corresponding fitted surface roughness of TiN thin films. Generally speaking, the variation in the surface roughness of thin films is related to film thickness, and their correlation can be attributed to the alteration of the internal microstructure of the films caused by varying film thickness [15]. Notably, two inflection points are observed in the roughness variation depicted in Figure 1b, potentially associated with crystallization phenomena occurring within these thin films.

### 3.2. GIXRD—Microstructure

**Figure 2:**
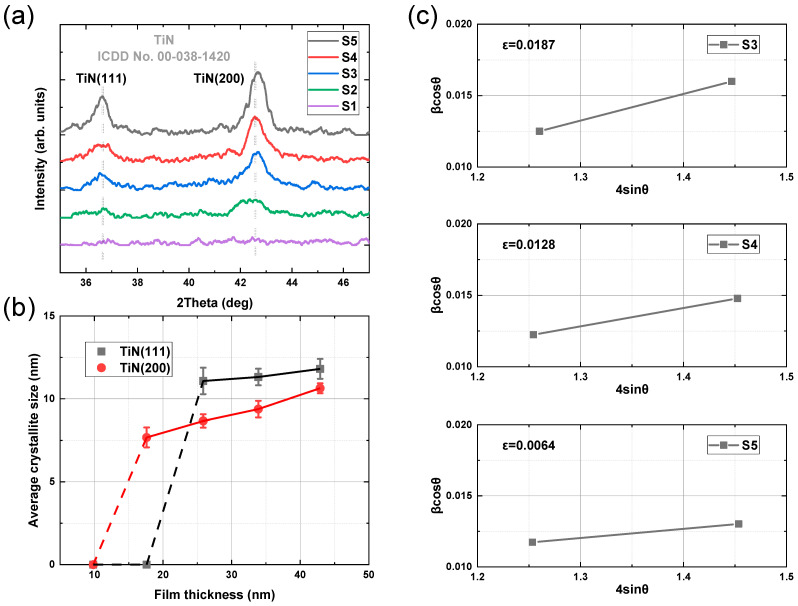
(**a**) GIXRD patterns of TiN thin films with varying thicknesses. (**b**) Average crystallite size of TiN thin films with varying thicknesses at (111) and (200) crystal orientation. (**c**) Microstrain obtained by Williamson–Hall plot of samples S3, S4, and S5.

Figure 2a shows the crystal structure and the preferred orientation of samples with varying thicknesses, which were characterized by GIXRD. According to the standard card ICDD No. 00-038-1420, the diffraction peak positions of TiN(111) and TiN(200) phases are 36.663° and 42.597°, respectively. In Figure 2a, there is no diffraction peak in the curve of sample S1 with the smallest film thickness, which proves that it is amorphous. With the increase in film thickness, sample S2 exhibits a (200) preferred orientation, and then samples S3, S4, and S5 change to a mixed (111)–(200) orientation. Liang et al. reported that TiN films prepared by DC sputtering exhibited a mixed (111)–(200) orientation within the thickness range of 14 nm to 40 nm [15]. In contrast, our study identified and explained the transition from an amorphous structure to a (200) orientation between 9.8 nm and 17.6 nm, followed by a transition from (200) to a mixed (111)–(200) orientation as the film thickness increased from 17.6 nm to 25.9 nm.

Pelleg et al. [22], U. C. Oh, and Jung Ho Je [23,24] proposed that the competition between strain and surface energies dictates the preferred orientation of thin films. For TiN with a small film thickness, the (200) orientation is favored due to lower surface energy, but as thickness increases, strain energy dominates, leading to a (111) orientation. However, this theory is more applicable to film growth conditions where thermodynamics, rather than kinetics, control texture formation, such as in films grown at high temperatures. Since most practical TiN deposition occurs at temperatures below 450 °C, the texture evolution is predominantly controlled by kinetic factors. According to the research of Gall et al. [25], by comparing the surface energies of N- and Ti-terminated TiN(111) surfaces, it is shown that the N-terminated surface is favored. Consequently, during the growth of TiN films, due to the lower chemical potential of Ti atoms on TiN(111) ($\mu_{Ti,111}=-7.80\;eV$) compared to TiN(200) ($\mu_{Ti,200}=-4.42\;eV$) (111)-oriented grains tend to grow more rapidly during polycrystalline TiN deposition. This results in the reduction in (200)-oriented grain width as the film thickness increases, leading to the gradual evolution of the TiN film towards a (111) orientation.

The average crystallite size of TiN can be calculated using the fundamental Debye Scherrer equation [26,27] based on the full width at half maximum (FWHM) and position of the diffraction peaks, as illustrated in Figure 2b.

(1) $$D=\frac{K\lambda }{\beta \cos\theta }$$

where D is the crystallite size in nm, *β* is the FWHM of the diffraction peak, *θ* is the Bragg’s angle, and *K* is the Scherrer constant, which relates to the form and size distribution of the crystallites. For TiN, due to its face-centered cubic (FCC) crystal structure, the commonly used value of the form factor *K* when estimating the crystallite size using Scherrer’s formula is 0.9.

The film thickness transition from 9.8 nm to 17.6 nm corresponds to a transformation from an amorphous state to (200)-oriented crystallization. Consequently, roughness substantially increases, corresponding to the first inflection point in Figure 1b. The microstructure of (111)-oriented films exhibits a triangular pyramid, while (200)-oriented films present a columnar microstructure, with the former contributing significantly to the surface roughness of the films [28]. As the film thickness changes from 17.6 nm to 25.9 nm, the dominance of the (200) orientation results in a relatively slow increase in film roughness. At 25.9 nm, the emergence of the (111) orientation is noted, corresponding to the second inflection point in Figure 1b. Thereafter, the average crystallite size of the (111) orientation rises proportionally with the film thickness, consistently surpassing that of the (200) orientation. Therefore, the film roughness is predominantly influenced by the (111) orientation when the film thickness changes from 25.9 nm to 42.9 nm, leading to a more pronounced increase in roughness. In addition, Tarniowy et al. found that amorphous TiN is less resistant to oxidation than the crystalline one [29]; therefore, the TiN films with a thickness of more than 17.6 nm have a more stable structure.

The Williamson–Hall (W-H) method is a technique used to analyze the peak broadening in X-ray diffraction (XRD) spectra, which is mainly used to separate the diffraction peak broadening caused by crystallite size and lattice microstrain [27,30,31]. This method can help determine the microstrain in the lattice. The basic formula for the Williamson–Hall method is as follows:

(2) $$\beta \cos\theta =\frac{K\lambda }{D}+4\varepsilon \sin\theta$$

where ε is the microstrain in the TiN films, *D* is the crystallite size, *K* is the Scherrer constant, and β is the FWHM. Due to the poor crystallization of samples S1 and S2, only the microstrain of samples S3, S4, and S5 can be calculated using the W-H method, as shown in Figure 2c. In this plot, the slope represents the microstrain. The microstrain in the TiN films with thickness changes from 25.9 nm to 42.9 nm decreased from 0.0187 to 0.0064.

### 3.3. XPS Depth Profile—Depth Distribution of Elements

**Figure 3:**
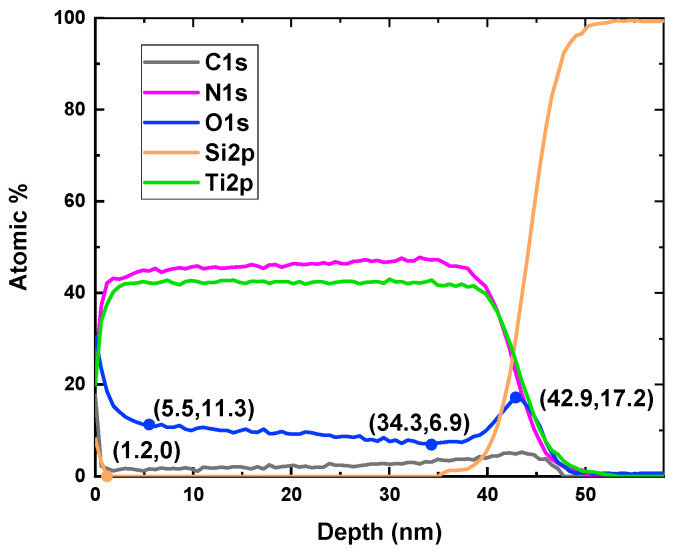
XPS depth profile showing the proportion distribution of C, N, O, Si, and Ti elements along the film depth.

In the process of GIXRR fitting, we found that the density of TiN thin films changes with depth. To explain this change, we selected the thickest sample with a film thickness of 42.9 nm and analyzed the proportion distribution of C, N, O, Si, and Ti elements along the depth by XPS depth profile. The step size of the analysis was 1 nm and the etching rate was calibrated using a standard SiO_2_ sample. The presence of a small amount of C may be due to carbon pollution during deposition, such as residual pump-line oil pollution, or carbon already present in the XPS system. Figure 3 shows that the proportion distribution of N, O, Si, and Ti varied quickly near the surface and the Si substrate, and the variation tendency leveled off from the depth of 5.5 nm to 34.3 nm. The reason was that TiO_2_ formed when TiN produced a chemical reaction with oxygen in the air and oxygen atoms attached to the Si substrate [32]. Since the top layer of Si may come from contaminants that adhere to the surface of the sample, the depth of 1.2 nm was regarded as the top surface of TiN, where the Si atoms disappeared, as shown in Figure 3. Similarly, the bottom surface of TiN could be determined by the maximum point of O with the depth of 42.9 nm.

**Figure 4:**
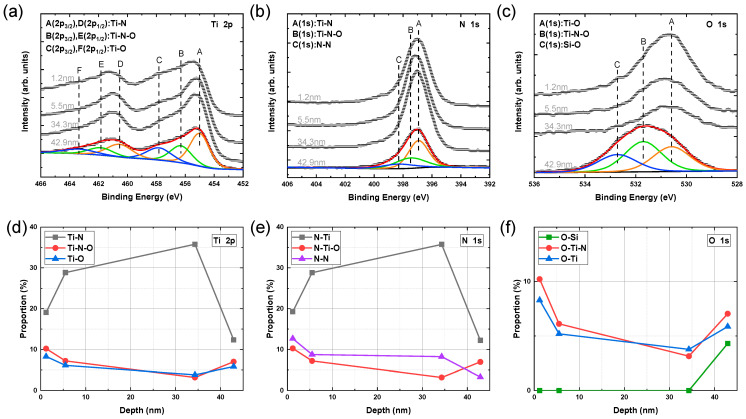
(**a**) Ti 2p, (**b**) N 1s, and (**c**) O 1s photoelectron spectra obtained at the excitation energy of 1000 eV from the TiN thin films. Chemical bond proportion distribution of (**d**) Ti 2p, (**e**) N 1s, and (**f**) O 1s along the depth.

Nevertheless, the previous examination of the XPS depth profile did not provide insights into the chemical states of individual elements. To address this limitation, the photoelectron spectra of Ti, N, and O were analyzed at several typical depth positions. This analysis could determine the chemical bond proportion, offering a more comprehensive understanding of the composition and bonding properties. In Figure 4a, the Ti 2p spectra can be assigned to three chemical bonds, Ti-N, Ti-N-O, and Ti-O, and the 2p orbit of each chemical bond split into two orbits of Ti 2p_3/2_ and Ti 2p_1/2_ [33,34]. The binding energies of Ti 2p_3/2_ for the three chemical bonds are 455.0 eV, 456.5 eV, and 458.0 eV, respectively [35]. And there is a 5.54 eV binding energy increase from Ti 2p_3/2_ to Ti 2p_1/2_. By multi-peak Gaussian fitting of Ti 2p spectra, the atomic proportion of each chemical bond in Figure 4d could be derived from the area proportion of the corresponding peaks. Meanwhile, the photoelectron spectra of N and O were analyzed in the same way in Figure 4b,c,e,f to show the result of atomic proportion. The binding energies of N 1s for the N-Ti, N-Ti-O, and N-N chemical bonds are 396.8 eV, 397.4 eV, and 398.1 eV, and the binding energies of O 1s for the O-Si, O-Ti-N, and O-Ti chemical bonds are 532.7 eV, 531.5 eV, and 530.5 eV.

As a result, the atomic proportion and its change trend were similar for the same bond in the analysis of different elements. Ti-N dominated the chemical bonds of Ti and N in all depth positions, and the atomic proportion of Ti-N decreased a little at the top and bottom surfaces of TiN film due to oxidation. From the top to the bottom of the TiN film, Ti-N-O and Ti-O showed a trend of first decreasing and then increasing, and Ti-N-O changed faster than Ti-O. The N-N showed an overall declining trend with a faster decrease in the top and bottom layers, while it tended to level off in the middle layer. Meanwhile, Si-O from the silicon substrate only appeared at the bottom surface of the TiN film.

The principle of reactive magnetron sputtering deposition of TIN film is as follows [36]:

$$N_{2}\to N_{2}^{+}+e\;\;(\mathrm{in}\;\mathrm{the}\;\mathrm{gas})\;N_{2}^{+}+e\to 2N\;\;(\mathrm{on}\;\mathrm{the}\;\mathrm{substrate})\;Ti+N\to TiN\;\;(\mathrm{on}\;\mathrm{the}\;\mathrm{substrate})$$

N_2_ played a key role in the plasma reactions during this process.

Through the analysis of photoelectron spectra of Ti, N, and O, it was found that there were five kinds of chemical bonds in the TiN film: Ti-N, Ti-N-O, Ti-O, N-N, and O-Si. From the interface position at a depth of 42.9 nm to a depth of 34.3 nm, Si-O bonds and free O atoms existed because some oxygen atoms were adsorbed onto the substrate. At the beginning of the deposition, Ti-N, Ti-N-O, and Ti-O would form simultaneously because the three elements, Ti, N, and O, were present in similar proportions. The Ti atom was more likely to combine with the N atom to form Ti-N due to the lowest binding energy of Ti-N bond, so Ti-N occupied the largest proportion. In the deposition process of the film, the oxygen atoms from the substrate were gradually consumed, so Ti-N-O and Ti-O were gradually reduced, and Ti-N was gradually increased.

At the depth of 34.3 nm, the proportion of N atoms was greater than O atoms, and the non-stoichiometric TiN_x_ film had structural defects, which easily led to the oxidation of the film [37]. Because there was some residual oxygen in the sputtering cavity, the O atom would gradually combine with Ti and N, which caused the proportion of the O atom to increase and the proportion of the N atom to decrease until the Ti/N proportion was close to 1 at a depth of 5.5 nm.

The result of XRD analysis showed that only the TiN phase was detected in the phase composition of the film, indicating that the film was still a TiN crystal structure. The N atom in TiN can be replaced by the O atom in any proportion to form a continuous solid solution TiON because the lattice parameters of TiN and TiO are very close [38]. Since the sample had been placed in the atmosphere for more than a month before XPS testing, the O atoms on the surface of the film came from the air, which gradually infiltrated from the surface into the film [39].

We conducted an XPS depth profile analysis to investigate the chemical composition at the interface between TiN and the substrate, as well as at the TiN–air interface. TiN is expected to be used as a protective layer to enhance the reusability of the mandrel in future mandrel–mirror separation processes. Therefore, it is crucial to examine both the bonding between TiN and the substrate and the potential interaction between TiN and optical reflecting film materials. This analysis will inform the selection of appropriate reflecting film materials or the addition of an interlayer for mirror separation to ensure that TiN remains stable on the mandrel surface while enabling smooth separation from the optical reflecting film, without causing damage to the TiN layer.

Consequently, our study aims to explore the elemental depth distribution of TiN films within the tens-of-nanometer scale. This investigation supports the validation of the stratified GIXRR fitting results for TiN presented in this paper and provides a foundation for future research. In contrast, the XPS depth profile analysis in previous studies, such as the 140 nm TiN film analyzed in Ref. [15], has primarily focused on relatively thick films but have failed to address the TiN–substrate interface. Therefore, our work is essential for filling this gap in understanding.

### 3.4. GIXRR Fitting—Film Density

As illustrated in Figure 3, the interior of this film primarily comprised elements such as C, N, O, and Ti. Consequently, chemical substances such as C, TiO_2_, and TiN, along with their respective densities, should be taken into account in the GIXRR fitting. The bulk densities of C, TiO_2_, and TiN are 3.52 g/cm^3^, 4.25 g/cm^3^, and 5.40 g/cm^3^, respectively.

Considering the XPS depth profile revealing a changing proportion distribution of C, N, O, and Ti elements along the film depth, the film density exhibits variation with depth. Based on GIXRD analysis, the TiN films in this study formed a polycrystalline state, which is more porous than bulk material, resulting in the fitted film density being lower than that of the bulk material.

According to XPS analysis, the top surface of the film had minor contamination of C, and the top and bottom layers contained higher levels of oxygen and lower levels of nitrogen compared to the middle layer.

**Figure 5:**
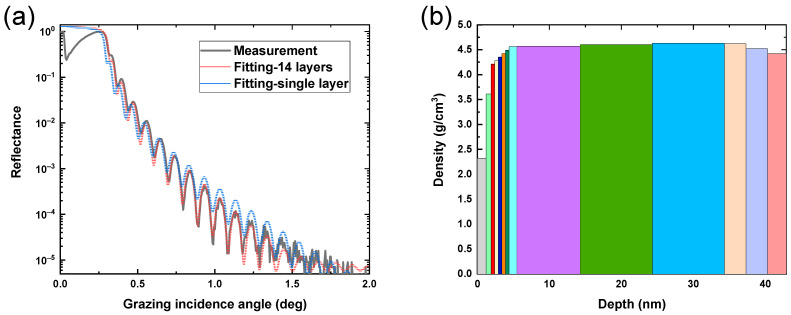
(**a**) GIXRR measurement curve and fitting curve of TiN thin film with a thickness of 42.9 nm. (**b**) The density of TiN thin film fitted along the depth by using a genetic algorithm. The film is divided into 14 layers, which are distinguished by different colors.

Based on the rate of change in element proportions across different regions, the fitting model of the film is divided into 14 layers with varying density and a total thickness of 42.9 nm on the silicon substrate to fit the GIXRR measurement curve, as shown in Figure 5a. The fitting curve exhibits improved conformity to the measurement curve compared to the results of the single-layer model. Since the density of TiN is higher than that of TiO_2_, the film density initially increases with an augmentation of N and a reduction in O. Conversely, the film density decreases in the bottom layer, and the rate of change in density aligns with the rate of change in the element proportion distribution.

### 3.5. AFM—Surface Morphology

**Figure 6:**
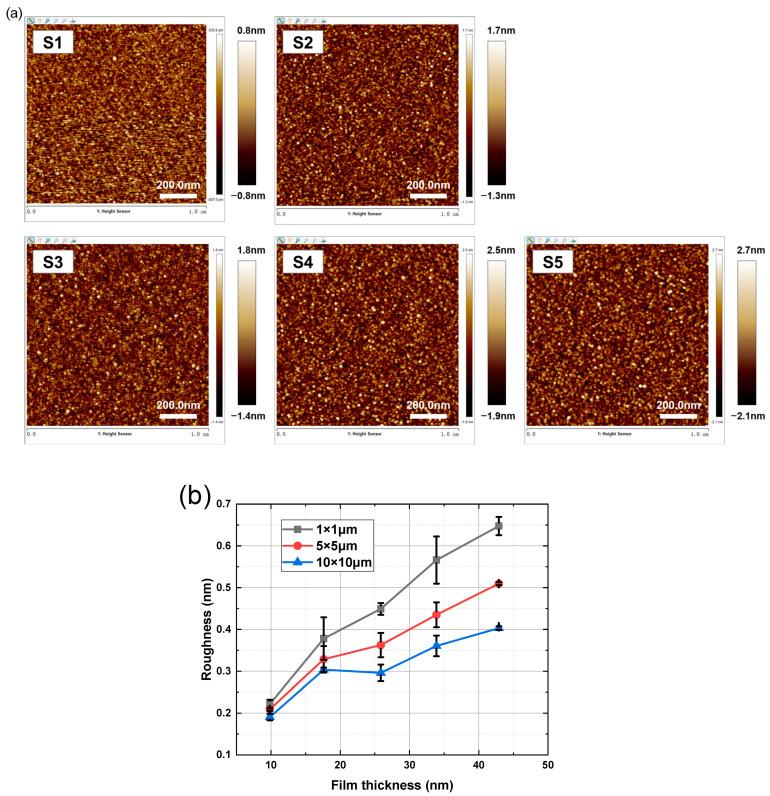
(**a**) AFM images of TiN films with varying thicknesses under 1 μm × 1 μm scan size. (**b**) Surface roughness of TiN thin films with varying thicknesses measured by AFM under different scan sizes.

The roughness of the TiN films with varying thicknesses was characterized by the RMS of AFM images under different scan sizes. Figure 6a shows the AFM images of five samples of TiN film with thicknesses varying from 42.9 nm to 9.8 nm under a scan size of 1 μm × 1 μm, whose roughness values are summarized using a black line in Figure 6b. The roughness of TiN film under scan sizes of 5 μm × 5 μm and 10 μm × 10 μm was also derived from AFM images, and are plotted in Figure 6b. The roughness of TiN film increases with an increase in film thickness in all scan sizes. Combined with the fitted results of GIXRR in Figure 1b, it is obvious that the increasing trend of sample roughness is consistent. In particular, the larger the scanning range, the smaller the roughness in the AFM test. Since AFM images are composed of 256 × 256 lines of pixels, the scale that can be resolved is related to the scan size, of which the scan size of 1 μm × 1 μm corresponds to a resolution of several nanometers. Therefore, film crystallization contributes more to the roughness of the smaller scan size due to the nanometer-scale average crystallite size of the sample (Figure 2b).

### 3.6. Intrinsic Stress

**Figure 7:**
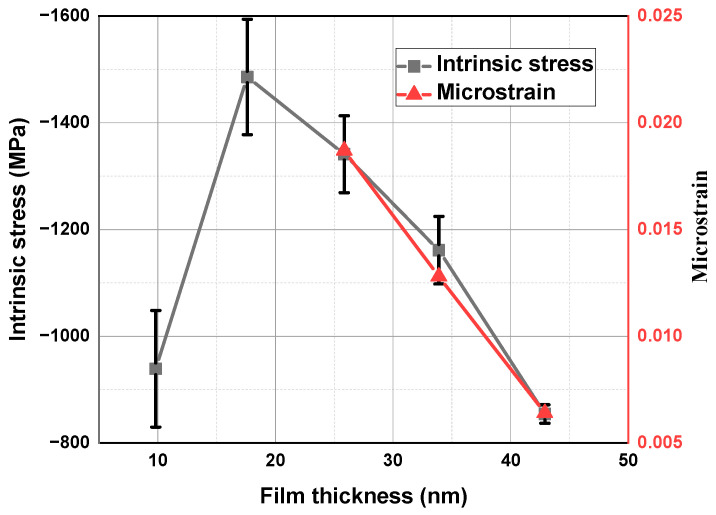
Intrinsic stress and microstrain of TiN thin films with varying thicknesses.

Figure 7 illustrates the intrinsic stress of the five samples and the microstrain values obtained for samples S3, S4, and S5 using the Williamson–Hall method. All samples exhibited compressive stress. For TiN films with thicknesses ranging from 25.9 nm to 42.9 nm, the trends in microstrain and stress are consistent, which can verify the reliability of the test. The thinnest TiN film exhibited a low compressive stress of 938.9 MPa, and the stress grew to 1485.9 MPa when the film started to crystallize at a thickness of 17.6 nm. After that, the compressive stress of the film began to decrease linearly with the increase in film thickness. According to Figure 6, the abrupt change in the stress curve is due to the transition of the film from amorphous to crystalline. According to the thin film island growth theory [40], during the initial nucleation and growth stages of thin film deposition, no intrinsic stress is generated because both the islands themselves and the atoms within the islands can move easily. As the island grows, crystals begin to appear in the film. At this time, the adhesion of the film to the substrate inhibits the expansion of the lattice, which makes the compressive stress increase significantly. After that, with the increase in film thickness, crystallite size increases, and crystallite boundary density decreases, which results in a decrease in film porosity. The lower the crystallite boundary density, the less likely it is that additional atoms will be inserted into the crystallite boundary, which reduces the compressive stress [41].

## 4. Conclusions

TiN thin films deposited at proportionally increased times ranging from 7.5 min to 37 min were fabricated to comprehensively analyze the evolution in thickness, microstructure, depth distribution of elements, surface morphology, and intrinsic stress. GIXRR curve fitting results show that the film thickness increases proportionally with the deposition time, which ranges from 9.8 nm to 42.9 nm. Interestingly, there are two inflection points in the curve of roughness increasing with film thickness. According to the XRD measurements, the TiN transforms from an amorphous state to (200) and (111)–(200) mixed-oriented crystallization, which accords with the energy minimization theory. Due to the greater contribution of the (111) crystal orientation to the film’s roughness compared to the (200) crystal orientation, the variation in crystallite size of the film explains the inflection point observed in the fitted Film thickness–roughness variation curve. According to the analysis results of XPS, there are Ti-N, Ti-N-O, and Ti-O chemical bonds inside the film, and the proportions of the three change with the depth of the film, which is due to the presence of O in the substrate, sputtering cavity, and air. In particular, the fitting of the GIXRR curve can be optimized by dividing the TiN layer according to the analysis results of XPS, and the fitted density of thin films is consistent with the variation in element distribution with depth. The fitting curve exhibits improved conformity to the measurement curve compared to the results of the single-layer model. The roughness of AFM is consistent with the trend of GIXRR fitting results. Due to the alignment of the sample’s average crystallite size with the resolution of a 1 μm × 1 μm scanning range, the crystalline structure of the film contributes more significantly to surface roughness at smaller scanning scales. According to the film island growth theory, the adhesion between the substrate and the film obstructs the expansion of the film lattice, resulting in the intrinsic stress of the film. With the increase in film thickness, the crystallite size increases, which leads to a reduction in intrinsic stress. Due to the impact of changes in thin film thickness on physical properties such as roughness, crystallinity, and stress, a comprehensive consideration of these physical properties should be considered when selecting the thickness of thin films for practical applications. As a release layer, TiN film should possess high surface quality, low intrinsic stress, and a stable crystalline structure. Therefore, following a comprehensive analysis, a TiN film with a thickness of approximately 25 nm is considered suitable for application as a release layer. The corresponding crystal phase is (111)–(200) mixed orientation, the surface roughness is about 0.4 nm, and the intrinsic stress is −1341.0 MPa.

## Figures and Tables

**Table 1:** Summary of relevant research on TiN films.

Researchers	Deposition Method	Film Thickness/nm
Hailong Liang et al. [15]	DC	7–300
J.M. Poitevin et al. [16]	DC	150
M.K. Hibbs et al. [17]	DC	4000
Tung-Sheng Yeh et al. [13]	Pulsed DC	500–1000
N.A. Richter et al. [18]	Pulsed DC	700
Udaiyappan Suresh et al. [19]	Pulsed DC	550

**Table 2:** Deposition time and fitted results of each sample.

Sample No.	Deposition Time/min	Film Thickness/nm	Surface Roughness/nm
S1	7.5	9.8	0.23
S2	15.0	17.6	0.41
S3	22.5	25.9	0.45
S4	30.0	33.9	0.56
S5	37.5	42.9	0.68

## Data Availability

The raw data supporting the conclusions of this article will be made available by the authors on request.
